# Adverse social behaviour at work and health-related employment exit: a prospective population-based four-wave survey

**DOI:** 10.1093/eurpub/ckac179

**Published:** 2022-12-07

**Authors:** Tom Sterud, Andrea R Marti, Eirik M Degerud

**Affiliations:** Department of Occupational Health Surveillance, National Institute of Occupational Health, Oslo, Norway; Department of Occupational Health Surveillance, National Institute of Occupational Health, Oslo, Norway; Department of Occupational Health Surveillance, National Institute of Occupational Health, Oslo, Norway

## Abstract

**Background:**

The level of evidence for various aspects of adverse social behaviour (ASB) at work as risk factors for exit from employment due to health problems or diseases is inconclusive.

**Methods:**

We obtained data from four consecutive surveys (2006/09/13/16) of the general population of Norway. Respondents who were interviewed in two consecutive surveys and employed at the first survey time point constituted the sample (*n* = 17 110 observations). We investigated associations of self-reported exposure to ASB (i.e. experiencing sexual harassment, bullying or violence/threats in the first survey) and health-related employment exit (i.e. individuals reporting exit from employment due to health problems or disease between two consecutive surveys) by means of mixed-effect logistic regression.

**Results:**

The prevalence of ASB and health-related employment exit was 10.8% (*n* = 1853) and 2.6% (*n* = 440), respectively. Adjusted for age, sex, level of education, occupation and weekly work hours, sexual harassment, bullying and violence/threats were associated with an increased risk of exit from employment. The odds ratios (ORs) for the association between exposure to any of the three aspects of ASB and employment exit was 1.78 [95% confidence interval (CI) 1.33–2.38]; the estimated corresponding population attributable risk was PAR% = 7.32 [95% CI 2.67–12.27]. Further adjustment of mental distress attenuated the observed association between exposure to any ASB and exit from employment (OR = 1.45 [95% CI 1.07–1.95], i.e. a reduction of 42% in the OR).

**Conclusions:**

ASB at work increases the risk of health-related exit from employment in the Norwegian workforce.

## Introduction

Health-related exit from employment implies that an individual must partially or completely exit from current employment because of reduced work capacity caused by mental or physical illnesses and injuries.^1^^,^^2^ The unemployment period following the exit may be temporary and lead to reemployment, or a permanent and final stage where the individual retires completely from working life.^3^^,^^4^ The ageing of the population has made extended working life a policy priority in Europe,^5^ and it is important to increase our knowledge of factors that increase the risk of early health-related exit from work and that are amenable to change. The causes of an early exit from employment are multifactorial and are not associated with medical conditions alone,^6^ and working conditions can play an important role in the part of the process related to the deterioration of health and the inability to cope with work demands.^7^^,^^8^ Mental disorders are among the leading causes of disability benefits worldwide,^9^^,^^10^ as well as in Nordic countries,^11–13^ and given the large body of research that recognizes psychosocial stressors at work as frequent and modifiable risk factors for common mental disorders^14^ and absence from work due to a mental disorder,^9^ the workplace constitutes an important arena where preventive efforts can be made. The most recent systematic review on the topic of psychosocial work conditions and the risk of disability retirement concluded that we need to know more about specific job stressors that can affect this risk and be applicable to interventions.^8^

During recent years, an increasing number of prospective studies have substantiated the importance of different aspects of adverse social behaviour (ASB), defined as any act of physical and verbal violence and intimidation at work,^15^ as risk factors for mental distress^16–22^ and sickness absence^23–26^ in the working population. However, based on a handful of studies from Scandinavian countries, the level of evidence for various aspects of ASB at work as risk factors for health-related exit from employment, often measured as disability pension, appears to be inconclusive. A rather large study (*n* = 12 303 individuals and *n* = 553 cases with disability pension) of a convenience sample of Norwegian employees, from different industries, reports an association between bullying and disability pension that was attenuated and not significant when adjusted for the level of education,^27^ while two studies of samples from the Danish working population reported mixed results for an association between bullying and disability pension; a positive association was reported in the study that combined data from four existing Danish cohort studies (*n* = 24 538/513 cases),^12^ while no association was observed in a smaller representative sample that was stratified by sex (*n* = 2567 women and 2794 men).^28^ The latter study reported a crude association between exposure and an excess risk of disability pension in women. However, the number of cases was low (*n* = 56 women and 31 for men) and the confidence intervals (CIs) were wide, making the estimates uncertain for women, and no estimates were reported for men.^28^ In contrast, in a large study that included multiple surveys of the Swedish working population (*n* = 66 253 individuals and *n* = 4062 cases), both sexual harassment and violence were significantly associated with the risk of disability pension.^29^

In summary, some large-scale studies report prospective associations between health-related working life exit and bullying^12^^,^^27^ and sexual harassment and violence,^29^ respectively. With few exceptions,^28^ these different forms of negative workplace behaviour are rarely examined together in the literature.^30^ Therefore, the objective of this study was to examine single and combined measures of ASB (i.e. bullying, sexual harassment and violence/threats) and their association with subsequent self-reported employment exit due to health problems or disease. We utilize longitudinal data on representative samples of Norwegian workers, including four consecutive surveys covering a period of 10 years. This allowed us to study the association between sexual harassment, bullying and threats/violence and a low prevalent outcome variable. Furthermore, in line with the literature showing a connection between ASB and mental distress, our objective was to assess whether the association between ASB and health-related employment exit is mediated by mental distress.

## Methods

### Sample description

The Survey of Level of Living-Working Conditions is an ongoing representative and longitudinal survey (i.e. all persons in the gross sample are invited to participate in at least three consecutive surveys) of Norwegian residents (i.e. a person is registered as a resident when he/she has taken up residence or intend to stay here for at least 6 months), aged 18–66 years. The survey is carried out by Statistics Norway according to the statutory rules. Respondents gave their informed consent prior to inclusion in the study. We combined data from four consecutive surveys: 2006, 2009, 2013 and 2016. The number of respondents in each survey, which includes both unemployed and employed respondents, ranged from 10 665 to 12 550; the response percentage ranged from 52.6% to 67%. In the present study, we include respondents who were interviewed in two consecutive surveys and who were defined as employed in the first of the two consecutive surveys. The number of individuals who participated in each of the consecutive surveys was *n* = 7559 (2006 and 2009), *n* = 6477 (2009 and 2013) and *n* = 3985 (2013 and 2016). The number of respondents interviewed in one, two and three surveys was 4060, 3343 and 2425, respectively, constituting a total of 10 029 individuals and 18 021 observations (table 1).

**Table 1:** Description of the full sample and criteria for inclusion in the present study

	Subsample 1	Subsample 2	Subsample 3	Total sample	
	Baseline: 2006 Follow-up: 2009	Baseline: 2009 Follow-up: 2013	Baseline: 2013 Follow-up: 2016	Total observations	Total respondents
Invited at baseline^a^	18 679	20 136	20 492	59 307	
Responded at baseline (*n* and %)	12 550 (67)	12 255 (61)	10 875 (53)	35 680	17 324
Responded at baseline and follow-up (*n* and %)	9371 (75)^b^	7837 (71)^b^	4774^c^ (66)^b^	21 982	13 465
Working population^d^	7559	6477	3985	18 021	10 029
Final sample^e^	7155	6173	3798	17 110	9468^f^

a Randomly drawn population sample in 2006, 2009 and 2013, respectively.

b Response percentage at follow-up among respondents at baseline (for subsample 3, the one-third of the respondents in 2013 who were not eligible at follow-up in 2016 was subtracted before calculating the response percentage).

c There were fewer respondents eligible for repeated measurements in subsamples 3 than in subsamples 1 and 2 as only two-thirds of the 2013 respondents were invited to the follow-up survey in 2016 due to a planned rotation of the panel (i.e. 7197 of 10 875).

d Working population sample = respondents who were in paid work during the interview week or were temporarily absent from such work at baseline and participated at follow-up. Cases at follow-up were in paid work at baseline and were no longer employed due to health problems at follow-up.

e Deleting missing values (i.e. respondents that were self-employed with no employees and respondents with missing values on either of the exposure variables or with missing values on education level or occupation).

f Sum of respondent that were interviewed about working conditions in one survey (*n* = 4060), two surveys (*n* = 3343) and three surveys (*n* = 2425).

### Outcome: health-related employment exit

Health-related exit from employment was determined by questionnaire at the time of follow-up and defined as respondents who were no longer employed at the time of the follow-up survey and who reported having quit their previous job due to their own health problems/their own disease based on the following question: ‘What was the most important reason for quitting your previous job?’.

### Exposure: ASBs

The items covering the different facets of ASBs were originally developed by a Nordic expert group,^31^ and have later been slightly modified. Sexual harassment (one item): Do you sometimes: (Q1) receive unwanted sexual attention, comments, etc., at your workplace? (Answer categories: yes, ≥1 a week; yes, ≥1 a month; no). Bullying (two items): ‘Do you sometimes: (Q1) get bothered or teased in an unpleasant way by your colleagues? (Q2) Get bothered or teased in an unpleasant way by superiors? (Answer categories: yes, ≥1 a week; yes, ≥1 a month; no). Threats/acts of violence (three items): ‘In the last 12 months: (Q1) Have you been the victim of violence at work that caused visible marks or physical damage? (Q2) Have you been the victim of violence at work that did not cause visible marks? (Q3) Have you been threatened at work in a way that you felt scared?’ (Answer categories: yes and no). We computed each factor into one dichotomous variable (yes for any item = 1, no = 0). Combined adverse social behaviour [ASB, combined] was defined as any exposure to ASB (yes in any element = 1, no = 0).

### Covariates

The educational level was based on administrative register data (based on the highest completed level of education) and coded into four educational levels (see table 2). Sex and age were based on self-reported information.

**Table 2:** Prevalence of adverse social behaviour and health-related employment exit by covariates

Covariates	Observations	Sexual harassment	Bullying	Violence/threats	ASB. combined	Health-related employment exit
	*N*	%	%	%	%	%
Total	17 110	3.7	2.7	6.5	10.8	2.6
Sex		*χ* ^2^ = 253.55 (1)*	*χ* ^2^ = 0.17 (2)^NS^	*χ* ^2^ = 174.3 (1)*	*χ* ^2^ = 270.6 (1)*	*χ* ^2^ = 27.0 (1)*
Men	8678	1.4	2.7	4.0	7.0	1.9
Women	8432	6.0	2.8	9.0	14.8	3.2
Age groups		*χ* ^2^ = 146.5 (2)*	*χ* ^2^ = 0.83 (2) ^NS^	*χ* ^2^ = 15.09 (2)*	*χ* ^2^ = 60.7 (2)*	*χ* ^2^ = 146.2 (2)*
17–34	4461	6.3	2.9	7.4	13.4	1.1
35–49	6981	3.6	2.7	6.7	11.0	1.9
50–66	5668	1.7	2.6	5.5	8.6	4.6
Education level		*χ* ^2^ = 28.0 (3)*	*χ* ^2^ = 20.2 (3)*	*χ* ^2^ = 127.6 (3)*	*χ* ^2^ = 95.3 (3)*	
Elementary level	1894	4.4	4.0	5.1	10.7	4.6
Upper secondary education	7700	3.6	2.9	5.6	10.2	3.4
University/college 4 years	5544	4.2	2.4	9.3	13.5	1.5
University/college 4 years+	1972	1.8	1.9	3.0	5.8	0.7
Occupations (ISCO code)		*χ* ^2^ = 540.7(16)*	*χ* ^2^ = 51.0 (16)*	*χ* ^2^ = 991.1 (16)*	*χ* ^2^ = 914.4 (16)*	*χ* ^2^ = 182.9 (16)*
Managers (11–14)	1775	1.1	2.1	3.4	5.8	1.3
Professionals (21, 24–26)	2149	1.9	2.1	1.9	5.2	0.9
Health professionals (22)	615	9.6	2.4	15.8	21.8	1.1
Teaching professionals (23)	756	2.0	3.0	6.6	10.3	1.9
Technicians, associate professionals (31, 33–35)	3775	2.0	2.3	6.6	9.8	1.6
Health associate professionals (32)	673	11.1	2.1	19.8	27.0	1.9
Clerks (41)	830	2.7	2.8	2.2	6.6	3.9
Customer services clerks (42–44)	253	2.8	3.2	3.6	8.7	2.8
Personal service workers (51)	1880	8.7	2.6	16.0	22.5	4.7
Sales workers (52)	956	6.4	3.0	2.8	9.7	3.5
Personal care workers (53)	268	12.7	5.6	25.0	33.6	2.6
Protective services workers (54)	33	21.2	9.1	12.1	30.3	6.1
Agricultural/forestry/fishery workers (61–64)	203	1.0	3.4	2.5	5.4	3.0
Craft and related trades workers (71–75)	1428	1.1	3.1	0.7	3.9	3.2
Plant-/machine operators, assemblers (81–83)	870	2.0	5.1	2.4	8.0	4.5
Elementary occupations (91–96)	393	3.3	4.6	2.8	7.6	9.2
Unspecified	253	2.8	0.8	2.8	5.9	2.8
Working hours		*χ* ^2^ = 115.9(3)*	*χ* ^2^ = 0.14 (3)^NS^	*χ* ^2^ = 88.8 (3)*	*χ* ^2^ = 107.5 (3)*	*χ* ^2^ = 182.9 (16)*
<10 h per week	645	7.0	2.6	7.1	13.2	5.9
10–20 h per week	1229	7.2	2.8	8.8	15.2	8.1
20–33 h per week	1941	6.0	2.8	10.8	16.1	3.5
>33 h per week	13 295	2.9	2.7	5.6	9.5	1.8
Anxiety depression		*χ* ^2^ = 131.1 (2)*	*χ* ^2^ = 327.8 (2)*	*χ* ^2^ = 80.2 (2)*	*χ* ^2^ = 301.9 (2)*	*χ* ^2^ = 178.3 (2)*
No	14 617	3.1	1.9	5.8	9.3	2.0
Little afflicted	1927	6.3	6.2	9.3	17.8	4.9
Somewhat or severely afflicted	566	10.8	12.4	13.4	27.7	9.7

*N*, number of respondents; ASB combined, any exposure to ASBs (yes at any of the four items); *χ*^2^, chi-square test statistic (degrees of freedom); NS, not statistically significant.

* *P* ≤ 0.05.

Occupation was based on an open questionnaire and was coded by Statistics Norway into a professional title according to the International Standard Classification of Occupations (ISCO-08). We combined one- and two-digit codes and recoded into 17 occupational groups (see table 2).^23^

Mental distress was measured with two items that were developed for use in the survey of living conditions since 1989^31^: ‘Over the past month, have you been very afflicted, quite afflicted, slightly afflicted, or not bothered by (Q1) dejection or depression? or (Q2) nervousness, anxiety, or restlessness?’ Mental distress was defined as ‘not bothered’, ‘slightly afflicted’ or ‘quite/very afflicted’ on either of the two questions.

### Statistics

First, we evaluated the risk of attrition (i.e. non-response at follow-up) associated with the exposure and the outcome variables using mixed logistic regression and described attrition according to other covariates using χ^2^ tests (crude associations). Second, the distribution of health-related employment exit was described according to ASBs, and covariates and differences were tested using chi-square tests (table 2). In the main analyses, to assess the association between ASB and employment exit, we applied mixed-effect logistic regression analyses (i.e. repeated measures logistic regression) using the glmer command of the package lme4 in the statistical software R version 4.0.2, which is the recommended approach when analysing non-normal outcome variables that are clustered within units (in the current case repeated observations from the same individuals). We set a significance level of 0.05 and report prospective associations as odds ratios (OR) with 95% CIs. First, health-related employment exit was regressed on the specific factors defined as ASBs: sexual harassment, bullying, and threats/violence, in a time-lagged model that included the following covariates measured in the baseline survey: sex, age, level of education, occupation, and weekly working hours (table 3). Second, we computed the same model with a combined measure of exposure to any type of ASB at baseline (table 4). Based on this model, we calculated the population attributable risk percentage (PAR%) to provide a quantitative estimate of the proportion of health-related employment exit in the population that are attributable to any exposure to ASB. We used this global variable (i.e. exposed to at least one act of ASB) to obtain a statistically more robust estimate (i.e. a larger number of exposed cases) and to take into account that the different acts of ASB are correlated, so that we do not overestimate the PAR% attributed to ASB in the working population. The calculation was based on the standard formula Pd × ((OR 1)/OR), where Pd is the proportion of cases exposed to the risk factor in question. The lower and upper limits of the 95% CI for PAR% were calculated from the general formula of PAR% using the lower and upper limits of the 97.5% CI for Pd and OR.^32^ Furthermore, we introduced an additional adjustment for mental distress measured at baseline and calculated the percentage change in the OR for exposure to ASB using the formula: (OR _main model_ − OR _main model adjusted for mental distress_)/(OR _main model_ − 1) × 100, to quantify the proportion of the association between ASB and employment exit that can be mediated by mental distress.

**Table 3:** Multiple mixed logistic regression: health-related employment exit regressed on sexual harassment, bullying and violence/threats

	*N*	Cases *n* (%)	OR^a^	95% CI
Sexual harassment (ref = no)	16 477	412 (2.5)	1.00	
Exposed	633	28 (4.4)	2.15	1.36–3.40
Bullying (ref = no)	16 645	417 (2.5)	1.00	
Exposed	465	24 (5.2)	2.20	1.38–3.51
Violence/threats (ref = no)	16 002	402 (2.5)	1.00	
Exposed	1108	38 (3.4)	1.45	1.00–2.12

*N*, number of respondents; Cases *n* and (%), number and proportion of respondents with health-related employment exit, respectively.

a Adjusted for sex, age, weekly working hours, education level and occupation measured at the baseline survey.

**Table 4:** Multiple mixed logistic regression: health-related employment exit regressed on combined exposure to adverse social behaviour (with and without adjustment for mental distress)

			Main model^a^		Main model^a^ + adjustment for mental distress^b^	
	*N*	Cases *n* (%)	OR	95% CI	OR	95% CI
ASB, combined (ref = no)	15 257	368 (2.4)	1.00		1.00	
Exposed	1853	72 (3.9)	1.78	1.33–2.38	1.45	1.07–1.95
					
			PAR%	95% CI		
					
PAR% (ASB, combined)			7.32	2.67–12.12		

*N*, number of respondents; cases *n* and (%), number and proportion of respondents with health-related employment exit, respectively; ASB, combined, any exposure to ASB (yes at any of the three facets of adverse social behaviour); PAR%, population attributable risk percentage.

a Adjusted for sex, age, weekly working hours, education level and occupation measured at the baseline survey.

b Main model adjusted for mental distress at baseline.

## Results

### Analyses of attrition and missing values (not shown in a table)

Attrition, defined as a respondent who was in paid work at the time of the baseline survey and did not respond to the follow-up survey (*n* = 3436 (27.3%)), was not associated with exposure to ASB (OR = 1.04, 95% CI 0.94–1.15) or mental distress (OR 1.04, 95% CI 0.95–1.14) at baseline. The level of attrition was slightly higher among men (28.1% vs. 26.5% among women), higher in the youngest age groups (35.1% for the age 17–34 vs. 23.1 and 25.2 in the age groups 35–49 and 55–66, respectively) and among respondents with fewer years of education (40.1% for the basic school level vs. 19.2% for 4 years of university/college). Furthermore, among eligible respondents who participated in two consecutive surveys, we omitted respondents with missing values (*n* = 532 individuals and 910 observations). Missing values on exposure to any ASB (*n* = 109 respondents) were associated with a non-significant increase in the odds of working disability (OR adjusted for age and sex = 1.47 95% CI 0.90–2.41).

### Descriptive analyses of the final sample

The sample that was available for the analyses constituted 9468 individuals and a total of 17 110 observations. Table 2 shows the prevalence ASBs and health-related employment exit according to covariates. During the follow-up period, 2.6% (*n* = 439 individuals and 440 observations) reported that they had left their job due to a health problem. The prevalence increased with age, was higher among women than among men and increased with fewer years of education. This employment exit was also related to occupation [range: 0.9% (professionals) to 9.2% (elementary occupations)], fewer working hours per week and mental distress. Furthermore, we observed consistent associations between sexual harassment and violence/threats and all covariates included in the regression models. Bullying was associated with mental distress, education level, occupation, but not age, sex and working hours.

### Main analyses

Table 3 shows the association between different aspects of ASB at baseline: sexual harassment, bullying and threats/acts of violence and the risk of health-related employment exit at follow-up. In the mixed regression model (adjusted for age, sex, level of education, occupation and weekly working hours), the risk was statistically significantly higher among respondents who reported sexual harassment (OR = 2.15, 95% CI 1.36–3.40) and bullying (OR = 2.20, 95% CI 1.38–3.51). A weaker and borderline non-significant association was observed for violence/threats (OR = 1.45, 95% CI 1.00–2.12).

Table 4 shows that exposure to any of the three aspects of ASB (i.e. ASB, combined) was associated with a 1.78-fold (95% CI 1.33–2.38) increase in the odds of health-related employment exit. The estimated population risk of employment exit attributable to any ASB, was 7.32% (95% CI 2.67–12.12). When adjusted for baseline mental distress, the OR for ASB combined was 1.45 (95% CI 1.07–1.95), which implies a 42% reduction in the OR.

## Discussion

We investigated the role of different aspects of ASB at work as risk factors for exit from employment due to health problems or disease, in a randomly drawn sample of the general working population. Exposure to any type of ASB was associated with a statistically significant increase in the risk of subsequent employment exit, and this association appeared to be mediated in part by mental distress. About 11% of the sample was exposed to any type of ASB at work and the corresponding proportion of health-related employment exits attributable to ASB was about 7%. This indicates the potential importance of reducing ASB on the workplace to prevent exit from employment at the population level.

### Comparison with previous studies

A novel aspect of the present study was the possibility of examining the association between combined exposure to different aspects of ASB in the workplace and health-related exit from employment. Although bullying, sexual harassment and threats/violence share important conceptual similarities, such as subjective perception of the target, ambiguity of intent and violation of organizational norms, these different forms of negative workplace behaviour are usually examined separately in the literature.^30^ Furthermore, based on some studies conducted in Scandinavian countries, the level of evidence for different aspects of ASB at work as risk factors for exit from work due to disease or disability appears to be inconclusive for bullying^12^^,^^27^^,^^28^ and sexual harassment and violence.^28^^,^^29^ Our results contribute to the limited literature on this topic and show that sexual harassment and bullying are associated with a higher risk of health-related employment exit, while we observed a slightly weaker and borderline non-significant association with violence/threats. Our results, based on a representative population and a substantial number of observations, add to the literature by demonstrating a robust association after adjustment for educational level and occupational information. About 7% of the observed employment exits were estimated to be attributable to ASBs at the population level. In comparison, at the European level, the population risk of depression attributed to bullying has been estimated at about 6%. The variation in the attributable risk between the countries was 0.5–17.2%; the attributable risk for Norway was 8.8%.^33^ Even if the outcome variable was not the same in our study and in the European study,^33^ together, the studies highlight a significant potential to prevent mental health problems and exits from work if exposure to ASB is eliminated from work life. However, this interpretation assumes that the exposure–response relationship is causal, for which there is limited evidence. Moreover, the accuracy of the PAR depends on the completeness of the specified model, and despite thorough control of confounding factors in the fully adjusted model, we cannot rule out residual confounding.

Furthermore, we observed that the strength of the associations between exposure to ASB and health-related employment exit was substantially reduced when adjusted for mental distress. This is consistent with the inference that a potential causal effect of ASB is mediated, in part, by increased mental distress. This interpretation is consistent with the number of prospective studies in recent years that indicate that exposure to different aspects of ASB can be detrimental to mental health.^17^^,^^19–22^^,^^34^ Furthermore, in a sister article based on a different sample selection from the same surveys as in the present study, we observed that bullying and sexual harassment were independent predictors of mental health problems. However, the data also indicated possible reversed associations,^17^ and the inference that mental distress can precede bullying has some support in the literature.^22^ Since our design allowed only simultaneous measurement of work conditions and mental health, it precluded formal pathway analyses that could allow us to establish temporal relationships among covariates. Therefore, we cannot rule out the alternative interpretation that the association between ASB and health-related employment exit is confounded by the baseline mental distress state of the respondent. Plausible explanations for this interpretation have been discussed in the literature. Anxious or distressed employees can struggle in periods with social interactions and provoke behaviour in others that appears abusive or threatening.^35^ At the same time, people with high levels of mental distress can also tend to interpret statements and behaviour as hostile,^36^ and have less resources to deal with these situations constructively.

### Methodological considerations

Among the strengths of this study are the large national random sample and the repeated measurement design, which includes four surveys over a 10-year period. The surveys have acceptable response rates, and a non-response examination by Statistics Norway showed only minor differences when comparing non-responders and responders.^37^ Furthermore, our analyses did not detect any associations between exposure to ASB or mental distress at baseline and non-response at follow-up. Therefore, in general, there is limited reason to suspect that bias due to non-response influenced the observed results. However, the study also has limitations that should be considered. The study is based on self-reported data, and it is not possible to rule out bias due to imprecision in the measurement of the study variables. First, our exposure measures were initially developed by a Nordic expert group for use in a national survey,^31^ but their construct validity has not been extensively tested. The mode of data collection used in this study was a telephone interview. Compared to mailed questionnaires, telephone interviews could lead to an underestimation of both exposure and outcome, because telephone respondents can be apprehensive about the interviewer’s judgments, particularly when it comes to socially stigmatized actions, such as bullying or mental health problems.^38^ Second, in the literature on working conditions and health-related exit from employment, the outcome variable most studied is disability pension.^8^ Register data are often considered the gold standard, but self-reported measures, as used in the present study, is considered a fairly valid indicator of employment exit due to disease or disability.^1^^,^^39^ Furthermore, due to differences in national social security systems when it comes to eligibility rules for receiving a disability pension, self-reported measures of health-related employment exit may even be more comparable in some contexts.^39^ However, we were unable to distinguish between temporary and permanent employment exit, something that can limit the external validity of the results compared to previous studies that mostly used administrative data (i.e. disability pension) to measure health-related employment exit.^12,27–29^ However, although disability pension is a predominantly permanent outcome, it can also be temporary.^2^^,^^3^

## Conclusions

First, this study shows that exposure to different manifestations of ASB at the workplace, including sexual harassment, bullying and violence/threat, can increase the risk of health-related exit from employment in the general working population. A plausible mechanism that links different manifestations of ASB to employment exit is the development of anxiety and depression problems that can cause people to leave their jobs due to mental health problems. If health problems persist, it can be difficult to return to work, and the result may be temporary or permanent exit from work. However, even though the inference that this association is partially mediated by mental distress was supported by the data in the present, we could not rule out the possibility that existing health problems also predict subsequent exposure to ASB. Consequently, large-scale studies following the same individuals at several time points are needed to provide better indications of causality and plausible mechanisms. Second, our results highlight that the total impact of ASBs on health-related employment exit can be substantial, and, therefore, early identification and routines to deal with the types of behaviour in organizations are important. However, to develop robust and effective interventions, more research is welcomed on the antecedents of adverse social at the workplace in combination with more research-based knowledge on whether policies and competencies to prevent and manage ASB at work are successfully implemented in organizations.

## Data Availability

Statistics Norway has an established policy for data sharing. Requests for data (i.e. The Norwegian Survey on Living conditions working conditions) can be addressed to the Norwegian Centre for Research Data (https://nsd.no/).
